# Supplementary material for: Which COVID policies are most effective? A Bayesian analysis of COVID-19 by jurisdiction
Source: PLoS One. 2020 Dec 29;15(12):e0244177. doi: 10.1371/journal.pone.0244177 (PMC7771876; doi:10.1371/journal.pone.0244177)
Supplement: S1 Fig — Dots = reported; X = outlier; Solid lines = model fit; Dashed lines = 95% intervals. (DOCX) [file pone.0244177.s001.docx]

**Figures**
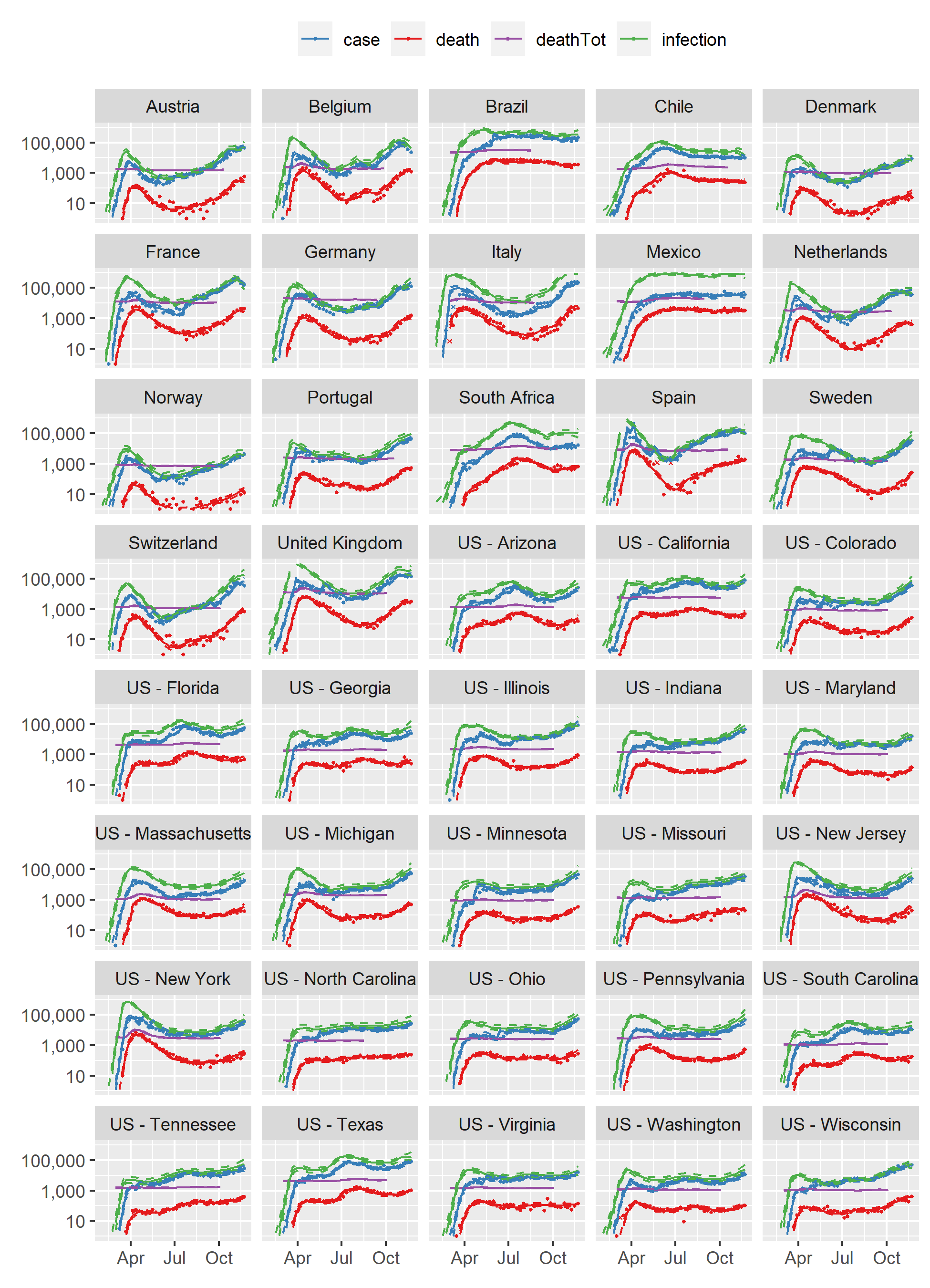
 **Figure S1.** Newly identified COVID cases, COVID deaths, total deaths, and infections per week (log scale). Dots = reported; X = outlier; Solid lines = model fit; Dashed lines = 95% intervals
