# Supplementary material for: Which COVID policies are most effective? A Bayesian analysis of COVID-19 by jurisdiction
Source: PLoS One. 2020 Dec 29;15(12):e0244177. doi: 10.1371/journal.pone.0244177 (PMC7771876; doi:10.1371/journal.pone.0244177)
Supplement: S2 Fig — Dots = median estimates; Lines = 95% intervals; Red = Base estimate outside of 95% posterior interval of changed specification. (DOCX) [file pone.0244177.s002.docx]

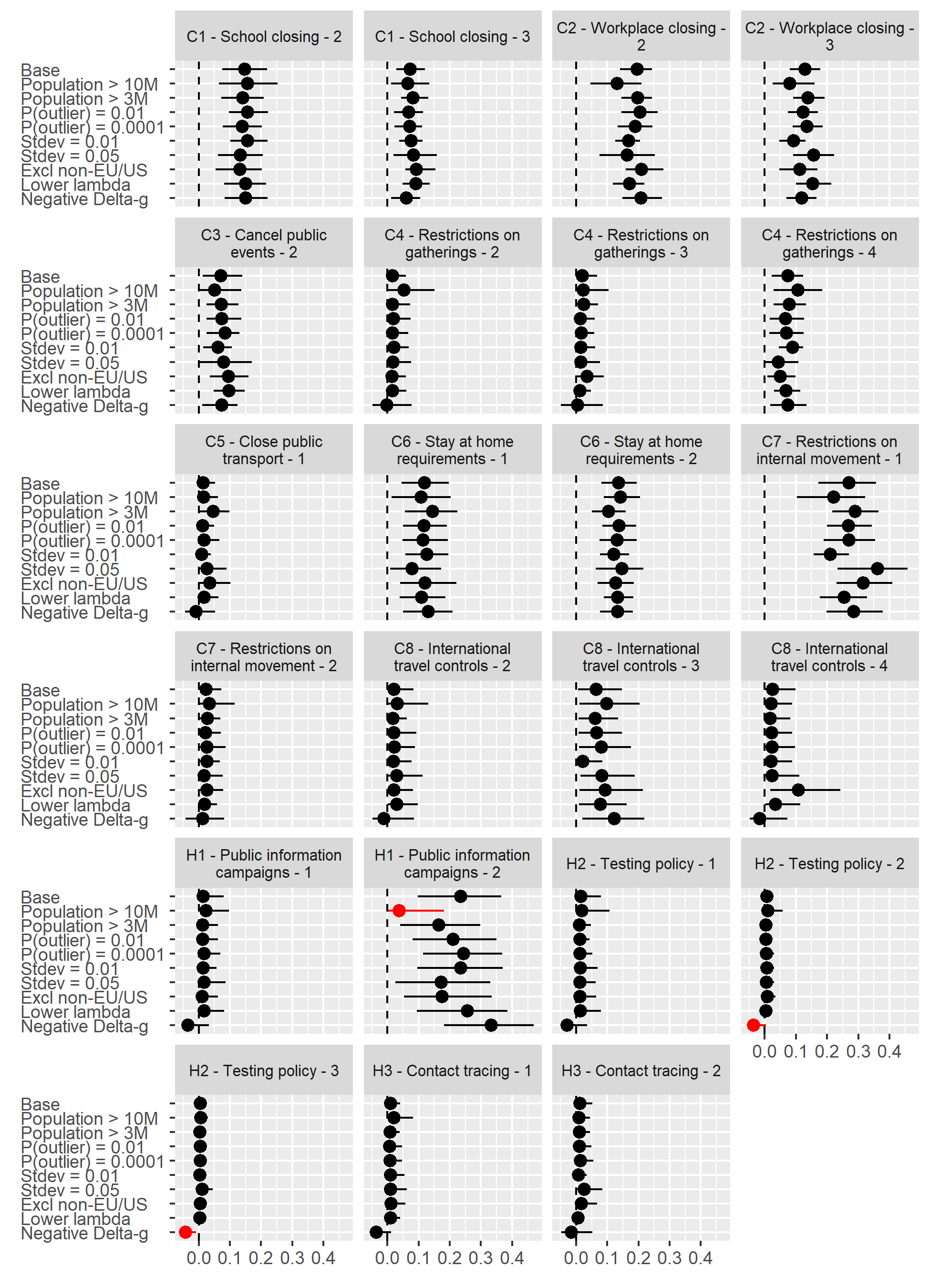


**Figure S2.**  Sensitivity of policy effect estimates (as reported in Figure 2 of the main text) to different model specifications. Dots = median estimates; Lines = 95% intervals; Red = Base estimate outside of 95% posterior interval of changed specification.
