# Supplementary material for: Which COVID policies are most effective? A Bayesian analysis of COVID-19 by jurisdiction
Source: PLoS One. 2020 Dec 29;15(12):e0244177. doi: 10.1371/journal.pone.0244177 (PMC7771876; doi:10.1371/journal.pone.0244177)
Supplement: S3 Fig — (DOCX) [file pone.0244177.s003.docx]

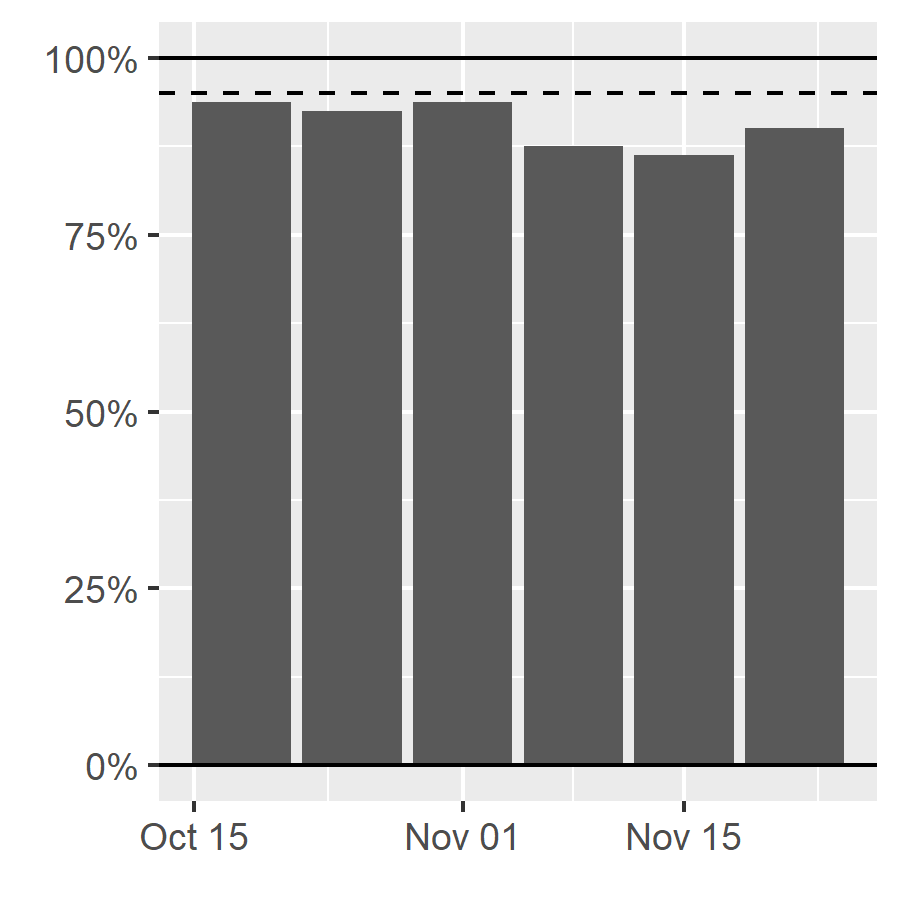


**Figure S3.** Percentage of weekly reported COVID cases and deaths within 95% interval of out-of-sample predictions from a model using input data up to 6 weeks prior to present.
