# Supplementary material for: Which COVID policies are most effective? A Bayesian analysis of COVID-19 by jurisdiction
Source: PLoS One. 2020 Dec 29;15(12):e0244177. doi: 10.1371/journal.pone.0244177 (PMC7771876; doi:10.1371/journal.pone.0244177)
Supplement: S4 Fig — Dots = reported; Solid lines = model prediction; Dashed lines = 95% intervals. (DOCX) [file pone.0244177.s004.docx]

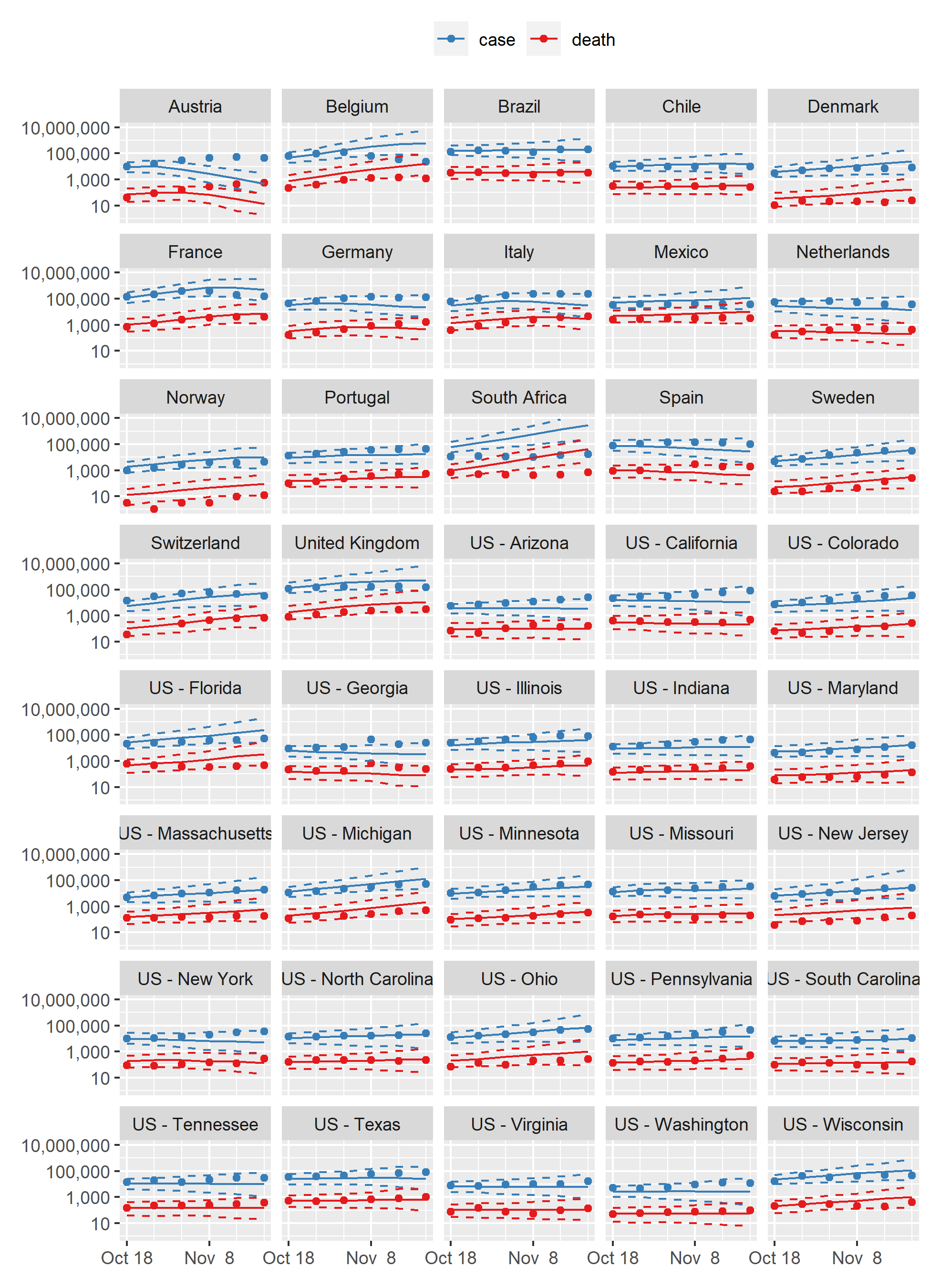


**Figure S4.** Out of sample estimates of newly identified COVID cases and deaths (log scale), with reported numbers. Dots = reported; Solid lines = model prediction; Dashed lines = 95% intervals
