# Supplementary material for: Which COVID policies are most effective? A Bayesian analysis of COVID-19 by jurisdiction
Source: PLoS One. 2020 Dec 29;15(12):e0244177. doi: 10.1371/journal.pone.0244177 (PMC7771876; doi:10.1371/journal.pone.0244177)
Supplement: S1 Table — (DOCX) [file pone.0244177.s005.docx]

**Table S1.** Posterior median and 95%-interval estimates for key variables of interest, as well as Bayesian estimate statistics *n*_eff_ and *R*_hat_.

|  | Median | 95% interval | | *n*_eff_ | *R*_hat_ |
| --- | --- | --- | --- | --- | --- |
| *w­*_1_^(case)^ | 0.974 | 0.875 | 0.999 | 771 | 1.00 |
| *w­*_2_^(case)^ | 0.026 | 0.001 | 0.125 | 771 | 1.00 |
| *w­*_1_^(death)^ | 0.096 | 0.071 | 0.122 | 715 | 1.00 |
| *w­*_2_^(death)^ | 0.438 | 0.376 | 0.508 | 430 | 1.01 |
| *w­*_3_^(death)^ | 0.318 | 0.236 | 0.395 | 729 | 1.00 |
| *w­*_4_^(death)^ | 0.149 | 0.086 | 0.204 | 665 | 1.00 |
| *φ*^(case)^ | 6.996 | 6.137 | 7.994 | 707 | 1.00 |
| *φ*^(death)^ | 7.447 | 6.285 | 8.866 | 712 | 1.00 |
| *φ*^(deathTot)^ | 345.9 | 307.8 | 390.7 | 594 | 1.00 |
