# Supplementary material for: Which COVID policies are most effective? A Bayesian analysis of COVID-19 by jurisdiction
Source: PLoS One. 2020 Dec 29;15(12):e0244177. doi: 10.1371/journal.pone.0244177 (PMC7771876; doi:10.1371/journal.pone.0244177)
Supplement: S2 Table — (DOCX) [file pone.0244177.s006.docx]

**Table S2**. Variance inflation factors for the policy fixed effects *δ_ipt_* by policy *p*.

| C1 - School closing - 2 | 3.05 |
| --- | --- |
| C1 - School closing - 3 | 1.91 |
| C2 - Workplace closing - 2 | 2.00 |
| C2 - Workplace closing - 3 | 1.91 |
| C3 - Cancel public events - 2 | 1.84 |
| C4 - Restrictions on gatherings - 2 | 5.19 |
| C4 - Restrictions on gatherings - 3 | 4.89 |
| C4 - Restrictions on gatherings - 4 | 1.57 |
| C5 - Close public transport - 1 | 1.53 |
| C6 - Stay at home requirements - 1 | 2.78 |
| C6 - Stay at home requirements - 2 | 2.06 |
| C7 - Restrictions on internal movement - 1 | 3.15 |
| C7 - Restrictions on internal movement - 2 | 1.55 |
| C8 - International travel controls - 2 | 2.58 |
| C8 - International travel controls - 3 | 2.79 |
| C8 - International travel controls - 4 | 1.19 |
| H1 - Public information campaigns - 1 | 2.16 |
| H1 - Public information campaigns - 2 | 3.80 |
| H2 - Testing policy - 1 | 1.79 |
| H2 - Testing policy - 2 | 2.51 |
| H2 - Testing policy - 3 | 1.57 |
| H3 - Contact tracing - 1 | 2.01 |
| H3 - Contact tracing - 2 | 1.67 |
| C1 - School closing - 2 | 3.05 |
| C1 - School closing - 3 | 1.91 |
